# Self-Care Practice and Associated Factors among Hypertensive Patients in Debre Tabor Referral Hospital, Northwest Ethiopia, 2020

**DOI:** 10.1155/2021/3570050

**Published:** 2021-08-11

**Authors:** Shegaw Gelaw, Melaku Kindie Yenit, Solomon Gedlu Nigatu

**Affiliations:** Department of Epidemiology and Biostatic, Collage of Medicine and Health Science, University of Gondar, Gondar, Ethiopia

## Abstract

**Background:**

Hypertension prevalence is continuously rising and is projected to be 1.56 billion cases by the year 2025. Despite the great progress made in the treatment of hypertension, many patients still do not achieve optimal results and experience devastating complications due to uncontrolled high blood pressure.

**Objective:**

The aim of this study is to assess self-care practice and associated factors among hypertensive patients.

**Methods:**

An institution-based cross-sectional study was conducted at Debre Tabor Referral Hospital, Northwest Ethiopia, from October to November 2020. A single population proportion formula and systematic random sampling technique was used to recruit 392 study participants. The data were entered to Epi-Info software version 7.1 and then exported to SPSS version 23 for analysis. A descriptive statistic was expressed as percentage, frequency, and mean. Finally, multivariable logistic regression was used to identify factors associated with dependent variable using a *p* value of <0.05.

**Results:**

A total 392 eligible hypertensive patients participated in the study. The self-care practice among hypertension patients was found to be 54.1%. Urban residency (AOR = 2.17; 95% CI, 1.2–3.9), social support (AOR = 2.12; 95% CI, 1.13–3.39), good knowledge (AOR = 1.83; 95% CI, 1.15–2.91), age between 40 and 64 (AOR = 3.15; 95% CI, 1.19–8.3), age ≥65 (AOR = 3.81; 95% CI, 1.35–10.7), and stress control (AOR = 1.6; 95% CI, 1.06–2.67) were predictors of hypertension self-care practice. *Conclusion and Recommendation*. The study revealed that almost one out of two hypertension patients had good hypertension self-care practice. Good social support, age greater than 40 years, urban residency, good basic knowledge, and having stress control were positively associated with hypertensive self-care practice.

## 1. Background

Hypertension is the increase in systemic blood pleasure (BP) of systolic blood pressure (SBP) above 140 mmHg or diastolic blood pressure (DBP) greater than 90 mmhg based on at least two separate readings on different occasions or 130/85 mmhg at home [1, 2].

According to the 2019 World Hypertension Report, an estimated 1.13 billion people have hypertension, more than two-thirds of whom lives in low- and middle-income countries [3]. Hypertension prevalence continues to rise and is projected to be 1.56 billion by 2025 [4]. In population-based studies and institution-based studies, its prevalence in Ethiopia was 9.3–30.3% and 7–17%, respectively [5]. According to the Ethiopian Federal Minster of Health (FMOH) 2019/20 annual report, out of the total screened adult population, 311,591 (12%) have raised BP. From these people who had raised BP, 132,777 (43%) had enrolled to receive care [6].

Untreated hypertension case causes a serious medical condition that increases the risk of hypertensive urgency and hypertensive emergency, which ends with heart attack, brain and kidney injury, and other cardiovascular diseases. Diseases like Angina, heart attack, and irregular heartbeat can also lead to a sudden death [3]. Hypertensive self-care practice is a strategy that describes how hypertensive individuals are monitoring and controlling their BP [7].

Hypertension self-care practice is a dynamic and active process required to control blood pressure based on knowledge, attitude, disciple, determination, commitment, self-regulation, empowerment, and self-efficacy [8]. It is essential for people who live with chronic conditions to have a better quality of life that prevents possible disability and reducing rising health expenditure. Having good hypertension self-care practice can reduce primary healthcare visit by 17% and emergency department visit by 50% [9]. One of the purposes of managing hypertension is to reduce BP by taking antihypertensive drugs [10].

The main hypertension self-care practice includes weight reduction to normal body weight, eating a diet rich in fruits and vegetables, dietary sodium restriction, engaging in regular aerobic activity such as brisk walking for at least 30 minutes per day in most days of the week, moderate alcohol consumption, and cessation of smoking [11].

Factors contributing to hypertension self-care practice were socioeconomic status, comorbidity, access to information, access to healthcare, number of medications, duration of treatment, age, gender, culture, educational status, and knowledge of the disease; self-efficacy, social support, and stress control were the major predicting factors towards adhering to hypertension self-care practice [12–17].

A previously done study in Ethiopia had some limitation. The existing studies miss some key variables: having BP apparatus and use of stress management [12, 16, 18, 19], self-care agency [12, 16, 19], social support [12, 19], and predominantly urban residents participating [18, 19]. Therefore, the aim of the study was to assess hypertension self-care practice and factors associated at Debre Tabor Referral Hospital in 2020.

## 2. Methods

### 2.1. Study Setting and Period

This study was conducted from October to November 2020, at Debre Tabor Referral Hospital (DTRH) in the chronic patient follow-up clinic. DTRH is located at the city of Debre Tabor, South Gondar administrative zone, Amhara National Regional State, Northwest Ethiopia, 667 km from Addis Ababa and 97 km from the city of Bahir Dar. It provides services to a population of 2,609,823 in the South Gondar area. It has one outpatient hypertensive clinic with a follow-up of about 880 hypertensive patients per month [20].

### 2.2. Study Design

An institution-based cross-sectional study was conducted.

### 2.3. Population

All adult hypertension patients with at least six months of follow-up at DTRH were the source population.

### 2.4. Sample Size Determination and Sampling Procedure

The sample size was determined by using a single population proportion formula by taking 44.7% proportion from previous study and assuming 5% marginal error, 95% confidence interval and 5% nonresponse, and the final sample size was 399 [21]. Systematic sampling technique was used to recruit study participants, and every other patient who came for follow-up in the hypertension chronic outpatient clinic was interviewed. The first patient was selected based on lottery method.

### 2.5. Data Collection Tools and Measurement

An interviewer gave structured questionnaire, and data abstraction format was used to collect data. The data were collected by three trained BSc holder nurses. It includes five parts, which were sociodemographic components, Hypertension Self-Care Activity Level Effects (H-SCALE), Appraisal of Self-Care Agency-Revised (ASAS-R), Multidimensional Scale of Perceived Social Support (MSPSS), Knowledge questions, and wealth index. Chart review was used to obtain BP current measurement and comorbidity.

#### 2.5.1. Hypertension Self-Care Activity Level Effects

H-SCALE is an outcome measurement that contains six subscales of self-care practice. These are low-salt adherence, weight management, physical activity, cessation of alcoholic, medication adherence, and cigarette smoking [22].

#### 2.5.2. Low-Salt Diet Adherence

Twelve items were used to assess low-salt diet adherence for the last seven days before the survey. Nine questions which are negatively framed questions, like eat smoked meats, add salt to your food at the table, and add salt to food when you are cooking and eat pickles and olives, were coded under reversed score. A mean score is calculated and equal to or greater than the mean score (6/12) which was considered as adherent to low-salt diet [13, 23].

#### 2.5.3. Physical Activity

Adults should accumulate at least 150 minutes of moderate intensity aerobic physical activity throughout the week or do at least 75 minutes of vigorous intensity aerobic physical activity throughout the week [13, 24]. Two items were used to assess physical activity as follows: “how many of the past 7 days did you do at least 30 minutes total of physical activity with moderate intensity?” “How many of the past 7 days did you do a specific exercise activity (such as walking) other than what you do around the house or as part of your work?” The response was summed from zero to 14. Participants adhere to physical exercise when the mean score was above 8 [22].

#### 2.5.4. Weight Management

It was measured using ten Likert-type scale items and used to assess weight control through diet control and physical exercise. Items assessed agreement with weight control activities in the last thirty days. Respondents who rated all the ten Likert-type scale items as either agree or strongly agree adhered to weight management [22]. Otherwise, they did not adhere to weight management.

#### 2.5.5. Smoking

Those who smoke cigarettes at least one puff per day or any amount of cigarette throughout the month were categorized under smokers, and those who did not smoke any number of cigarettes within a week were classified as nonsmokers. Respondents who reported 0 days of smoking cigarettes were considered nonsmokers [22].

#### 2.5.6. Alcohol Drinking

Those who drink any amount of alcohol, types of alcohol (traditional drinks like: teji, araki, filter, and tella), and any modern alcohol throughout the week will be assessed by three questions. “On average, how many days per week do you drink alcohol?” “On a typical day that you drink alcohol, how many drinks do you have?” And “what is the largest number of drinks that you have had on any given day within the last month?” Responses range from zero to 21. Participants who did not take any type and amount of alcohol in the last 7 days or who did not drink at all were considered abstainers [22].

#### 2.5.7. Medication Adherence

The hypertension patient should be able to use prescribed antihypertensive medication at the right time and with full dose [13, 25]. For these self-reported medication adherences, we used three items (taking blood pressure medication, taking it at the same time every day, and taking the recommended dosage) to assess the number of days in the last week (seven days). Responses were summed (range 0–21), and participants who were reported to follow these 3 recommendations on 7 out of 7 days were considered adherent (score = 21) [22].

#### 2.5.8. Social Support

A support given by an individual family or groups of people to hypertensive person is available in time of need. It could be psychological, physical, and financial help. It was measured by using a Multidimensional Scale of Perceived Social Support (MSPSS). MSPSS was assessed by 12 Likert scale questions with one to five responses from strongly disagree as one and strongly agree as five. Based on the mean score of 3.44, it can be categorized as good social support if it is above the mean else poor social support [26].

#### 2.5.9. Knowledge

Nine knowledge questions were used in assessment with yes/no responses. They are adopted from the knowledge of hypertension self-care practice on Addis Ababa [14, 27] by computing a median score from answering correctly with yes = 1 and no = 1 ranging from 1 to 9 with a median score of above 6 which was good knowledge [27]. Respondents were labeled to have knowledge on hypertension control and management if their score was equal to mean score or above on the closed ended knowledge questions about hypertension and self-care practice.

#### 2.5.10. Self-Efficacy

Self-efficacy is the person's sense of confidence or ability to conduct his/her own activity. It was measured using 15 questions of Appraisal of Self-Care Agency-Revised (ASAS-R), each individual question having one, strongly disagree, and five, strongly agree. The lack of time to take care of yourself and lack of energy were under reversed score. Based on the mean score (3.34), being equal to or above it is considered as good self-efficacy. Participants who were scored below the mean score were categorized as poor self-efficacy [28].

### 2.6. Operational Definitions

Operational definitions are given as follows:

1. Good self-care practice: a hypertension patient adhered to at least four out of six subscales of the H-SCALE [29].
2. Poor self-care practice: a hypertension patient adhered to more than two subscales of the H-SCALE.

### 2.7. Data Quality Control Measures

Both data collectors and supervisors were trained for one and half days on the objectives, tools, and data collection approach. The questionnaire was translated from English to Amharic language and translated back into English by another person to check for consistency. A pretest was conducted one week before the actual data collection among 5% (twenty-two) from the hypertensive treatment clinic at Adisalem Primary Hospital. During pretesting, the questionnaire was checked for its clarity, simplicity, understandability, consistency, and coherency. A reliability test (Cronbach alpha) was performed to check the internal consistency of with 89% for H-SCALE, 88.8% for ASAS-R, 92.2% for MSPSS, and 82% for knowledge on hypertension self-care.

### 2.8. Data Analysis and Processing

Data were checked, cleaned, and entered into the Epi-Info software version 7.1, and it was exported to the Statistical Package for Social Sciences (SPSS) version 25 software for analysis. Descriptive statistics, such as frequency, central tendency measurement, and dispersion measurement, were used to characterize the participants. The principal component analysis was used to compute the wealth status of the participants. The Hosmer and Lemeshow goodness-of-fit test for the model was also checked. Associations between independent variable and dependent variables were analyzed first using bivariable analysis to identify factors, which are significantly associated with the outcome variable when *p* value <0.2 with adjusted odds ratio (AOR) and a 95% confidence interval (CI). A multivariable analysis was done to declare significance at *p* value <0.05.

## 3. Result

### 3.1. Sociodemographic Characteristics

A total of 392 eligible hypertension patients who were attending chronic disease follow-up unit at DTRH were interviewed during the study period which gave a response rate of 98.2%. We could not incorporate seven study subjects because they were not willing to be part of this study. Of the total participants, 209 (53.3%) were males. The age category of 182 (46.4%) of the participants was above 65 years. The mean age of the study participants was found to be 61.8 years with ±14.1 standard deviation. About the marital status, the majority of the respondents were married, which accounts for 280 (71.4%). Almost half (50.8%) of the participants were categorized under poor by their wealth index (Table 1).

### 3.2. Clinical Characteristics

A hundred and seventy-six (44.9%) respondents were diagnosed during routine checkup and 149 (38%) were diagnosed after complaining from sign and symptoms. The remaining 67 (17.1%) participants also were diagnosed after they developed complications. The mean duration of hypertension was 4.25 years with a range of 0.8–20 years. Nearly half of the respondents (47.2%) took more than two types of antihypertensive medications.

The majority of the participants, 318 (81.1%), had normal BMI within the range of 18 to 24.9 kg/m^2^. A total of 119 (30.3%) respondents had medically confirmed comorbidity, largely heart diseases and diabetes mellitus, which accounts for the largest numbers with 28.8% and 24.8%, respectively. About 53.3% of the respondents controlled their hypertension, which is <140/90 mmHg, with the average 3 consecutive follow-ups, and 69.4% of them had a good hypertensive self-care practice (Table 2).

### 3.3. Behavioral Factors of Self-Care Practice

A total of 198 (50.5%) respondents had a good social support. Respondents having poor social support (66.7%) had good hypertension self-care practice. Self-care agency was determined by the 15-item questions with a mean of 3.34 ± 3.27 scores. Among the respondents, 204 (52%) of the hypertension patients had good self-care agency, and 126 (61.8%) of them had good self-care practice. Two hundred fourteen (54.6%) participants were used a stress reduction management. Of them, 51% talked with families or friends, 24.7% watched television or radio, 11% performed exercise, 11% took coffee or tea, and 1% read books (Table 3).

### 3.4. Knowledge on Hypertension Control and Management

Nearly 49% (95% CI: 43.5–53.4) of participants had good knowledge on hypertension control and management, and 34.6% of them had good self-care practice. Most (77.6%) of the participants know that meals rich in green bananas, baked chicken, and beans are good for a person with hypertension control, and 74% of them know drinking alcohol has a negative impact on persons with hypertension (Table 4).

### 3.5. Hypertension Self-Care Practice

A total of 212 hypertensive patients had a good hypertensive self-care practice computing from the medication adherence, low-salt adherence, physical exercise, weight management, cessation of alcoholic drinking, and smoking cigarette with a prevalence of 54.1% (95% CI: 49.1–59). Two hundred fifty-four (64.8%) respondents adhered to the recommended antihypertensive medication, and 32.1% of them did not adhere to low-salt diet. Among the hypertensive patients, 99% of them were nonsmokers, with not even a single puff of cigarette within a month (Table 5).

### 3.6. Factors Associated with Hypertension Self-Care Practice

Age, religion, educational status, marital status, residency, stress reduction management, number of medications, social support, appraisal of self-care agency, wealth index, and knowledge were significant in the bivariable logistic regression. However, in the multivariable logistic regression analysis, age greater than or equal to 65 and the age between 40 and 64 years, residency, stress management, social support, and knowledge were independent predictors of self-care practices.

The result of multivariable logistic regression analysis showed that odds of good self-care practice among ages within 40–64 and > 65 were 3.15 and 3.8 times more likely as compared to age less than 39 (AOR = 3.15; 95% CI: 1.19, 8.3 and AOR = 3.8; 95% CI: 1.35, 10.7, respectively). The odds of having good self-care practice among urban residency were 2.17 compared to rural residence (AOR = 2.17; 95% CI:1.21, 3.9). The odds of good self-care practice among hypertension patients who use stress management were increased by 60% as compared to their counterpart (AOR = 1.6; 95%CI: 1.06, 2.67). Getting good social support was 2.12 times more likely to have good hypertension self-care practice than those who get poor social support (AOR = 2.12; 95% CI: 1.13, 3.39). Having good knowledge on hypertension control and management increases the chance of good self-care practice by 83% as compared to having poor knowledge (AOR = 1.83; 95% CI: 1.15, 2.91) (Table 6).

## 4. Discussion

In this study, 54.1% of hypertension patients had good self-care practice towards hypertension. It is consistent with a study done in Addis Ababa (51%) [19] and Dessie Town (49%) [18]. On the contrary, self-care practice in this study is higher than studies conducted in Tigray with 23% [12] and in Harar town with 29% [16]. This variation might be that the participants in this study had relatively good basic knowledge regarding hypertension, and a large number of our study participants were urban residence which paves the way to accessible health facility easily. Low self-care practice is directly linked to uncontrolled hypertension. This is lower than the study conducted in Saudi Arabia (74.4%) [30] and India (81%) [31]. This variation could be due to a difference in the sociodemographic and socioeconomic status, which made good self-care practices and level of knowledge.

This study revealed that the odds of age greater than or equal to 65 years had 3.8 times more likely to have good self-care practice compared to age less than 40 years. This was supported by the study done in Harar town [16] and in Jima [21]. The study done in North Ethiopia Tigray; hypertension patients with the age of greater than or equal to 65 years were 3.2 times more likely to adapt to good self-care practice towards hypertension than those above the age of 65 years [12]. This variation may be because the government currently gives puts emphasis on NCD prevention and control and healthcare providers are given health education and training. However, the finding of our study contradicts with the results of a study conducted in Dessie Town. It showed that older age patients are less likely to have good self-care practice [18]. The reason could be due to the increased self-awareness and the need to control hypertension, where in turn patients were on follow-up for years and exposed to various instructions on hypertension.

In this study, the odds of living in urban area were two times more likely to have good self-care practice. This study was supported by the study conducted in Addis Ababa. Living in urban areas was three times more likely to adapt to good self-care practice [19]. The possible reason is that living in urban area was near information and media access to hypertension control and management. In this study, 55% of the urban participants had good knowledge.

In the current study, the odds of having good self-care practice towards hypertension were increased by 60% of those having stress control on hypertension compared to patients in the absence of stress control. Stress control is a positive predicting factor on the control of hypertension and self-care practice [32]. Some literatures showed that people used alcohol and smoke cigarette to relieve stress temporary. In this study, 56% of the participants were talking to their family and peers to relieve stress, and 54% of hypertensive patients had used such mechanisms as stress control. This is consistent with the fact that the study in Saudi Arabia with stress control (55.7%) was used [30]. This is relatively higher stress control compared to Brazil, where 39% of the hypertensive patients had stress control [33]. This difference might be the time to give more attention to NCD control and stress control becomes a one component of self-care practice to control BP [1]. Stress control is essentially reduced high BP through talking to the family or friends.

The study also revealed that the odds of having a good self-care practice was 2.2 times more likely among having good social support compared to poor social support. This is in line with a study done in Dessie town, Harar town, and China [16, 18, 34]. In a study done in Jimma social support had a strong effect on medication adherence and low-salt diet adherence [15]. The odds of having family social support in China increased by 39% for medication adherence [34]. This might be because having good social support is essential for hypertension self-care and enhancing team-based care and task shifting protocol [35]. In this context, social support is much stronger, and people had a good habit in discussing the social issue. The WHO self-care intervention guidelines support the current study that social support helps people cope better with everyday life and help beneficiaries to resume their normal lives [36].

The study revealed that 48.5% of the hypertensive patient had good basic knowledge regarding hypertension, which is similar to a study done in South Ethiopia, and 44% of participants have good knowledge regarding hypertension [37]. However, the finding of a study in Addis Ababa was 48.6%, which is supported in the current finding [14]. However, the study done in India on knowledge on hypertension self-care practice was much higher than the current study with 98% having good knowledge [38]. This variation might be the level of education and residency that are accessible to information.

The odds of having a good basic knowledge on hypertension control were 1.8 times more likely than poor basic knowledge to adhere self-care practice. This is consistent to the study conducted in Harar town [16] and in Tigray [12]. It is also in line with a similar study in Saudi Arabia [30]. The possible justification for this finding is having knowledge promote patients to give more emphasis on self-care practice. In this study, the majority of the participants had higher level of education. In addition, self-care can enhance the ability to cope to and comply with medical regimens and disease management. The current findings were supported by the study conducted in Southern Iran. Knowledge is a strong predictor for weight management and medication adherence [39].

Self-care practice for hypertension is an important strategy to improve long-term adherence of the recommended lifestyle changes [40]. This research has implication for researcher to figure out what is the consequence and time to develop adverse outcome among those who poorly practice self-care activities, and their number is significant. For a long time, Ethiopia implementing prevention led to health policy; it also gave a dual attention to noncommunicable diseases. So, this study will show to the policy maker where the country stands and what should be done.

## 5. Conclusion and Recommendation

The current study revealed that almost one out of two hypertension patients had good hypertension self-care practice in Debre Tabor Referral Hospital. Stress control, good basic knowledge about hypertension control and management, good social support, age greater than or equal to 40 years, and urban residency were positively associated with good self-care practice. Special attention should be given to young people who live with hypertension by giving a life skill training on how to manage their stress. The family should play indispensable role by proving social support for their family member who lives with hypertension. Health education should be continuing for hypertension patients which address basic knowledge about hypertension, how to control their BP, and teach that it is a lifelong disease. So, they should highly be advised to adhere to their antihypertension medication. The government should give a dual attention for rural residence through facilitating accessing information related to hypertension.

## Figures and Tables

**Table 1:** Sociodemographic characteristics of hypertensive patients in Debre Tabor Referral Hospital, Northwest Ethiopia, 2020.

Variable category	Self-care practice	
	Good *N* (%)	Poor *N* (%)
Age category		
≤39 years	10 (35.7%)	18 (64.3%)
40–64 years	104 (57.1%)	78 (42.9%)
≥65 years	98 (53.8%)	84 (46.2%)

Sex		
Male	114 (54.5%)	98 (53.6%)
Female	95 (45.5%)	85 (46.4%)

Marital status		
Single	16 (51.6%)	15 (48.4)
Married	162 (57.9%)	118 (42.1%)
Separated	34 (42%)	47 (58%)

Educational status		
Informal education	83 (47.7%)	91 (52.3%)
Primary education	36 (56.3%)	28 (43.7%)
Secondary education	22 (50%)	22 (50%)
Collage and above	71 (64.5%)	39 (35.5)

Residency		
Urban	175 (60.8%)	113 (39.2%)
Rural	37 (35.6%)	67 (64.4%)

Religion		
Orthodox	163 (50.9%)	157 (49.1%)
Muslim	36(63.2%)	21( 36.8%)
Protestant	13 (86.7%)	2 (13.3%)

Wealth index		
Rich	110 (57%)	83 (43%)
Poor	102 (51.3%)	97 (48.7%)

**Table 2:** The clinical related health profiles of hypertensive patient on Debre Tabor Referral Hospital, Northwest Ethiopia, 2020.

Variable category	Self-care practice	
	Good *N* (%)	Poor *N* (%)
First diagnosed		
During routine checkup	85 (48.3%)	91 (51.7%)
After complaint	89 (59.7%)	60 (40.3%)
After complication	53 (79.1%)	14 (20.9%)

Duration of illness		
≤4 years	131 (53.9%)	112 (46.1%)
4–8 years	65 (54.2%)	55 (45.8%)
>8 years	16 (55.2%)	13 (44.8%)

Presence of comorbidity		
Yes	68 (57.1%)	51 (42.9%)
No	144 (52.7%)	129 (47.3%)

BMI		
<18.5	2 (33.3%)	4 (66.7%)
18.5–24.9	180 (56.6%)	138 (43.4%)
≥25	30 (44.1%)	38 (55.9%)

Number of medications		
1 drug	85 (47.2%)	95 (52.8%)
2 drugs	108 (58.4%)	77 (41.6%)
3 drugs	19 (70.4%)	8 (29.6%)

BP apparatus		
Having BP	42 (77.8%)	12 (22.2%)
No BP	170 (50.3%)	168 (49.7%)

BP status		
Controlled	145 (69.4%)	64 (30.6%)
Uncontrolled	67 (36.6%)	116 (63.6%)

**Table 3:** The behavioral related health profiles of hypertensive patient on Debre Tabor Referral Hospital, Northwest Ethiopia, 2020.

Variable category	Self-care practice	
	Good *N* (%)	Poor *N* (%)
Chat chewing		
Chat chewer	12 (52.2%)	11 (47.8%)
Non-chat chewer	200 (54.2%)	169 (45.8%)

Social support		
Good social support	132 (66.7%)	66 (33.3%)
Poor social support	80 (41.2%)	114 (58.8%)

Self-care agency		
Good self-care agency	126 (61.8%)	78 (38.2%)
Poor self-care agency	86 (45.7%)	102 (54.3%)

Use stress management		
Yes	128 (59.8%)	86 (40.2%)
No	84 (47.2%)	94 (52.8%)

Frequency of health facility visit		
Once a month	201 (55.2%)	163 (44.8%)
Twice	11 (39.3%)	17 (60.7%)

**Table 4:** Level of knowledge on hypertension self-care of hypertensive patients on Debre Tabor Referral Hospital, Northwest Ethiopia, 2020.

Level of knowledge	Frequency (*N*)	Percentage
Hypertension is a serious condition that can lead to complications	274	69.9
Being overweight is risk to raise blood pressure	263	67.1
Salt consumption raises blood pressure	279	71.2
Physical exercise helps reduce blood pressure	243	62.0
Smoking cigarettes has a negative effect on persons with hypertension	249	63.5
Khat chewing has a negative effect on persons with hypertension	242	61.7
Drinking alcohol has a negative impact on persons with hypertension	290	74.0
A diet which contains fruits and vegetables is good for a person with hypertension	236	60.2
A meal rich in green bananas, baked chicken, and beans is good for a person with hypertension	304	77.6

**Table 5:** Self-care practices of hypertensive patients at Debre Tabor Referral Hospital, Northwest Ethiopia, 2020.

Variable	Category	Frequency	Percentage
Medication adherence	Adherent	254	64.8
	Not adherent	138	35.2

Physical activities	Adherent	203	51.8
	Not adherent	189	49.2

Weight management	Good weight management practice	216	55.1
	Poor weight management practice	176	44.9

Low-salt diet adherence	Poor adherent to low-salt diet	126	32.1
	Good adherent to low-salt diet	266	67.9

Cigarette smoking	Nonsmoker	382	97.4
	Smoker	10	2.6

Alcohol drinking	Abstain	257	65.6
	Not abstain	135	34.4

Self-care practice	Good self-care practice	212	54.1
	Poor self-care practice	180	45.9

**Table 6:** Factors associated with self-care practice of hypertensive patients in Debre Tabor Referral Hospital, Northwest Ethiopia, 2020 (*n* = 392).

Variables	Self-care practice		COR (95% CI)	AOR (95% CI)	*p* value
	Good *N* (%)	Poor *N* (%)			
Age category					
<39 years	10 (35.7%)	18 (64.3%)	1	1	
40–64 years	104 (57.1%)	78 (42.9%)	2.4 (1.05–5.48)	**3.15 (1.19-8.3)** ^*∗*^	0.021
≥65 years	98 (53.8%)	84 (46.2%)	2.1 (0.92–4.79)	**3.8 (1.35-10.7)** ^*∗*^	0.012

Religion					
Orthodox	163 (50.9%)	157 (49.1%)	1	1	
Muslim	36 (63.2%)	21 (36.8%)	1.65 (0.9–2.95)	0.61 (0.31–1.21)	0.204
Protestant	13 (86.7%)	2 (13.3%)	6.26 (1.4–28.2)	2.17 (0.51–9.35)	0.112

Marital status					
Single	16 (51.6%)	15 (48.4%)	1.48 (0.64–3.39)	1.65 (0.55–4.9)	0.545
Married	162 (57.9%)	118 (42.1%)	1.89 (1.15–3.13)	1.51 (0.8–2.8)	0.808
Separated	34 (42%)	47 (58%)	1	1	

Educational status					
Informal education	83 (47.7%)	91 (52.3%)	1	1	
Primary education	36 (56.3%)	28 (43.7%)	0.5 (0.3–0.82)	0.7 (0.37–1.31)	0.502
2^nd^ education	22 (50%)	22 (50%)	0.71 (0.37–1.33)	0.78 (0.38–1.57)	0.882
Collage and above	71 (64.5%)	39 (35.5%)	0.55 (0.27–1.12)	0.53 (0.23–1.24)	0.063

Residency					
Urban	175 (60.8%)	113 (39.2%)	2.8 (1.76–4.47)	**2.17 (1.21-3.9)** ^*∗*^	0.012
Rural	37 (35.6%)	67 (64.4%)	1	1	

Use stress management					
Yes	128 (59.8%)	86 (40.2%)	1.66 (1.12–2.49)	**1.6 (1.06-2.67)** ^*∗*^	0.03
No	84 (47.2%)	94 (52.8%)	1	1	

No. of medications					
1 drug	85 (47.2%)	95 (52.8%)	0.37 (0.15–0.9)	0.53 (0.2–1.38)	0.795
2 drugs	108 (58.4%)	77 (19.6%)	0.59 (0.24–1.4)	0.65 (0.25–1.69)	0.149
3 drugs	19 (70.4%)	8 (2%)	1	1	

Social support					
Good social support	132 (66.7%)	66 (33.3%)	2.85 (1.9–4.3)	**2.12 (1.3-3.39)** ^*∗*^	0.02
Poor social support	80 (41.2%)	114 (58.8%)	1	1	

Self-care agency					
Good self-care agency	126 (61.8%)	78 (38.2%)	1.91 (0.1.28–2.8)	1.35 (0.82–2.22)	0.21
Poor self-care agency	86 (45.7%)	102 (54.3%)	1	1	

Knowledge					
Poor knowledge	88 (43.6%)	114 (56.4%)	1	1	
Good knowledge	124 (65.3%)	66 (34.7%)	2.43 (1.6–3.66)	**1.83 (1.15-2.91)** ^*∗*^	0.01

Wealth index					
Rich	110 (57%)	83 (43%)	1.31 (0.88–1.95)	1.26 (0.80–2.0)	0.3
Poor	102 (51.3%)	97 (48.7%)	1	1	

COR: crude odds ratio; AOR: adjusted odds ratio; CI: confidence interval. Note: significance at *p* value <0.05.

## Data Availability

The datasets used and analyzed during this study are available from the corresponding author on reasonable request.
